# Endovascular Treatment of Ruptured Very Small Intracranial Aneurysms: Complications, Recurrence Rate, and Clinical Outcomes

**DOI:** 10.3389/fneur.2021.767649

**Published:** 2022-01-04

**Authors:** Feiyun Qin, Jiaqiang Liu, Xintong Zhao, Degang Wu, Niansheng Lai, Zihuan Zhang, Zhenbao Li

**Affiliations:** Department of Neurosurgery, The First Affiliated Hospital of Wannan Medical College (Yijishan Hospital), Wuhu, China

**Keywords:** very small intracranial aneurysms, ruptured, endovascular treatment, complications, recurrence, clinical outcomes

## Abstract

**Objective:** The aim of this study was to evaluate the safety and efficacy of endovascular treatment for ruptured very small (≤3 mm) intracranial aneurysms (VSIAs).

**Methods:** The clinical data and imaging results for 152 patients with VSIAs treated with coil embolization from August 2014 to June 2020 were retrospectively reviewed. The influential factors related to the preoperative complications, aneurysm recurrence, and clinical outcomes for these patients were analyzed.

**Results:** Among 152 patients with ruptured VSIAs, 90 were treated with coil embolization alone, while 62 were treated with stent-assisted coil embolization. Eighteen patients experienced intra and/or postoperative complications (overall incidence = 11.8%). One person died of intraoperative aneurysm re-rupture and postoperative rebleeding (mortality rate = 0.65%). Twenty patients had various degrees of neurological dysfunction (morbidity rate = 13.1%). Statistical analysis showed that there was no independent risk factor associated with perioperative complications. The rate of complete aneurysm occlusion at discharge and follow-up was 76.3 and 86.2%, respectively. A total of 105 patients underwent digital subtraction angiography during follow-up, and 18 of them experienced postoperative recurrence (recurrence rate = 17.1%). Seven patients were retreated (retreatment rate = 6.7%). The use of stents was the only factor that affected the postoperative recurrence of aneurysm. The incidence of favorable clinical outcomes (Glasgow Outcome Scale score ≥ 4) at discharge and follow-up was 86.2 and 97.1%, respectively. Univariate analysis showed that the preoperative Hunt-Hess grade, CT Fisher grade, and perioperative complications were risk factors for poor clinical outcomes. Multiple logistic regression analysis showed that perioperative complication was the most significant risk factor for the clinical prognosis of patients with ruptured VSIAs.

**Conclusion:** Endovascular treatment is a safe and efficient approach for ruptured VSIAs. Stent-assisted coiling reduced the recurrence rate of aneurysm without increasing the incidence of perioperative complications. The Hunt-Hess grade, CT Fisher grade, and perioperative complications were independent factors associated with the clinical outcomes of patients with ruptured VSIAs, and perioperative complication was the most significant risk factor for poor prognosis in patients.

## Introduction

Rupture of intracranial aneurysm is the most common cause of spontaneous subarachnoid hemorrhage with high mortality and mobility. With the advances in imaging technologies, especially the widespread use of three-dimensional (3D) digital subtraction angiography (DSA), more very small intracranial aneurysms (VSIAs) have been detected in clinical practice. Approximately 13.2–15.1% of intracranial aneurysms are VSIAs and ruptured VSIAs account for 6–7% of ruptured intracranial aneurysms (1–3). The prophylactic treatment for unruptured VSIAs is still controversial due to the low annual spontaneous rupture rate (4). On-demand treatment for ruptured VSIAs, however, is necessary.

Surgical clipping is a conventional treatment for intracranial aneurysm, but not an ideal approach for VSIAs because these aneurysms are small and often associated with a high incidence of complications, such as clip slippage, intraoperative rupture, intracranial infection, postoperative epilepsy, etc. (5, 6).

With the development of interventional materials and technologies, endovascular embolization has become the first-line treatment method for intracranial aneurysms. However, it is still challenging to place the coils into VSIAs, which are associated with a higher complication rate than larger aneurysms (7). In the present study, we evaluated the safety and efficacy of endovascular treatment for ruptured VSIAs by retrospectively analyzing the clinical data and imaging results of 152 patients with ruptured VSIAs treated with coil embolization. We further analyzed the influential factors associated with the perioperative complications, recurrence rate, and clinical outcomes for these patients.

## Methods

### Study Design

In this retrospective study, data for 152 patients who were hospitalized for ruptured VSIAs and treated with coil embolization were analyzed. The study protocol was approved by the Ethics Committee of the Yijishan Hospital Affiliated to Wannan Medical College. Written informed consent was waived due to the retrospective nature of the study.

The inclusion criteria were as follows: (1) patients with subarachnoid hemorrhage caused by ruptured aneurysms (maximal diameter ≤ 3 mm); (2) treated with coil embolization; and (3) had complete clinical data and imaging results. Patients who met the following criteria were excluded: (1) multiple intracranial aneurysms; (2) complicated with arteriovenous malformation, moyamoya disease, or arteriovenous fistula; and (3) infectious aneurysm, fusiform aneurysm, or blood blister-like aneurysm.

There were 1,070 patients with ruptured intracranial aneurysms treated with coil embolization in our department from August 2014 to June 2020. These patients comprised 152 with ruptured VSIAs and 918 with other ruptured aneurysms with a maximum diameter of >3 mm. The following clinical data were collected: age, sex, hypertension, diabetes, smoking history, aneurysm location, Hunt-Hess grade, CT Fisher grade before coiling, immediate and follow-up aneurysm occlusion, perioperative complication, and clinical outcomes at discharge and follow-up.

### Endovascular Procedure

All procedures were performed under general anesthesia and all patients were systemically heparinized. The femoral artery was punctured using the modified Seldinger technique. After successful sheath placement, cerebral angiography and 3D reconstruction were performed. A guiding catheter (5F or 6F guiding catheter or DA catheter) was placed in the bone segment of the internal carotid artery or in the V2 segment of the vertebral artery according to the vascular tortuosity of the vessels. The microcatheter was shaped according to the shape of the aneurysm and its relationship with the parent artery and inserted into the aneurysm cavity under the guidance of a guide wire. The coils were then filled. For patients who underwent stent-assisted coil embolization, the microcatheter and stent catheter were inserted into the aneurysm cavity and the distal end of the parent artery under the guidance of a guide wire, respectively. After the coil was partially filled, the stent was half released to cover the aneurysm neck, and the stent was completely released after the aneurysm was completely occluded.

If the aneurysm re-ruptured during the surgery, the coils were rapidly filled in until no further bleeding was observed. If the patient experienced acute thrombosis formation, tirofiban was given until the thrombosis dissolved or the artery recanalized.

For patients treated with stent-assisted coiling (SAC), a loading dose of 300 mg clopidogrel and 300 mg aspirin was administered orally or by a nasogastric tube before the procedure. After coiling, 75 mg of clopidogrel and 100 mg of aspirin were given daily for 6 weeks, followed by 100 mg of aspirin daily for at least 12 months. The response of each patient to antiplatelet agents was recorded and the medication regimen was adjusted according to the results of thromboelastography.

### Outcome Measures

The following outcomes were measured: immediate and follow-up angiographic results, perioperative complications, and clinical outcomes at discharge and follow-up. The degree of aneurysm occlusion was evaluated with the Raymond-Roy grade (8): grade I indicated complete occlusion (no contrast agents observed in the aneurysm), grade II indicated neck remnant (contrast agents observed in the aneurysmal neck), and grade III indicated partial occlusion (contrast agents observed in the aneurysmal cavity). Perioperative complications included intraprocedural rupture (IPR), acute thrombosis formation, coil migration, postoperative cerebral infarction, and aneurysm rebleeding. Clinical outcomes were assessed as follows using the Glasgow Outcome Scale (GOS): 1, death; 2, no response, unable to respond to the external environment; 3, able to move as ordered, but unable to live independently; 4, able to live independently, but unable to return to work or go to school; 5, able to return to work or go to school. A GOS score of ≥4 was defined as a good clinical outcome.

### Statistical Analysis

Statistical analysis was performed using the SPSS V.19.0 (IBM SPSS; Armonk, New York, USA). Data are presented as means ± standard deviation for quantitative parameters and as frequencies for categorical parameters. The risk factors for procedure-related complications, postoperative recurrence, and clinical outcomes were assessed by independent samples *t*-test, χ2 test, or Fisher's exact test. Factors with a *P*-value of < 0.1 in univariate analysis were analyzed by multivariate logistic regression analysis. The OR and 95% CI were calculated. A *P*-value of < 0.05 was considered statistically significant.

## Results

### Characteristics of Patients at Baseline

**Figure 1:**
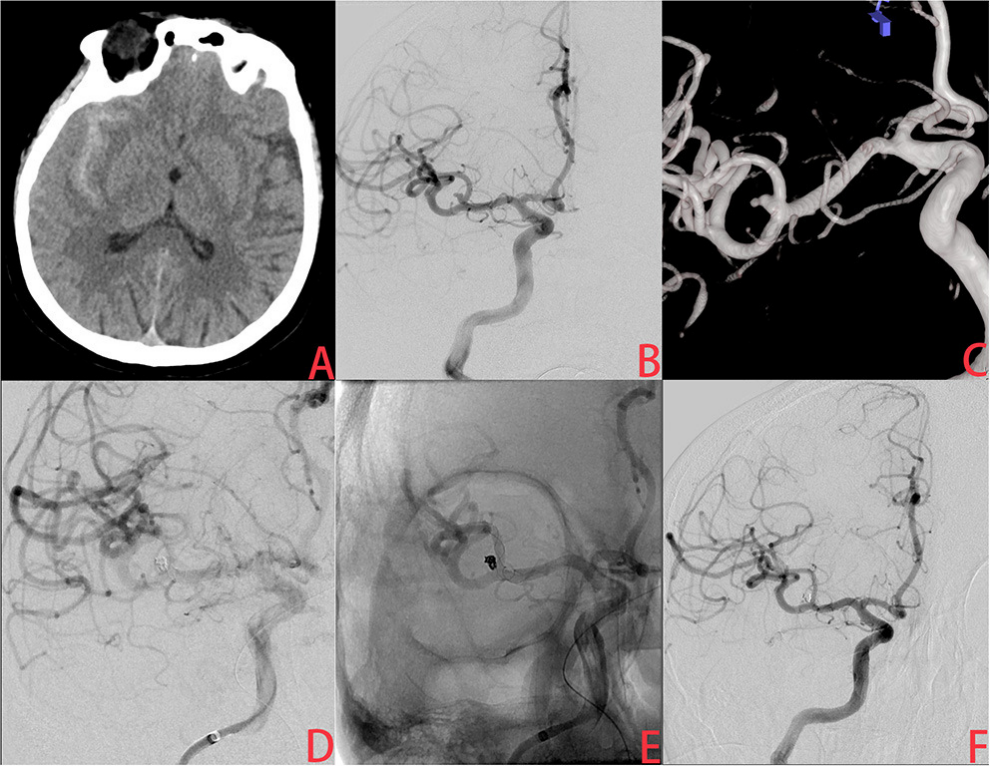
A ruptured very small middle cerebral artery (MCA) intracranial aneurysm treated with stent-assisted coiling (SAC). **(A)** The patient was admitted with spontaneous subarachnoid hemorrhage. **(B,C)** Cerebral angiography and 3D reconstruction revealed a tiny MCA aneurysm with a bleb at the tip. **(D,E)** The aneurysm was treated with stent-assisted coiling embolization using a Leo baby stent (2.5 × 18 mm). Immediate angiography showed that the aneurysm was completely occluded. **(F)** A year later, angiographic images showed complete occlusion of the aneurysm without in-stent artery stenosis.

A total of 152 patients with 152 ruptured VSIAs were included in this study. There were 98 (64.5%) women. The mean age of these patients was 56.2 ± 11.2 years (range 23–84) and the mean size of the aneurysms was 2.6 ± 0.4 mm (range 1.5–3.0). Of 152 patients, 62 (40.8%) were treated with SAC, while 90 (59.2%) were treated with coiling alone (CA, Figure 1), including one treated with balloon-assisted coiling. The baseline characteristics of the patients are shown in Table 1.

**Table 1:** Characteristics of patients at baseline.

**Characteristics**	
Age, mean ± SD, years	56.1 ± 11.2
Female, *n* (%)	98 (64.5)
Hypertension, *n* (%)	73 (48.0)
Diabetes, *n* (%)	7 (4.6)
Smoking history, *n* (%)	11 (7.2)
Hunt-Hess grade 1-2, *n* (%)	106 (69.7)
CT Fisher grade 1-2, *n* (%)	89 (58.5)
**Location**, ***n*** **(%)**	
AcomA	64 (42.1)
PcomA	27 (17.8)
MCA	15 (9.9)
A1 segment	9 (5.9)
Pericallosal artery	6 (3.9)
ICA	16 (10.5)
AchA	8 (5.3)
BA	2 (1.3)
Pca	2 (1.3)
Pica	3 (2.0)
Neck size, mean ± SD, mm	1.9 ± 0.5
Aneurysm size, mean ± SD, mm	2.5 ± 0.4
Neck-dome ratio, mean ± SD	0.90 ± 0.27
**Treatment method**, ***n*** **(%)**	
Coiling alone	90 (59.2)
Stent-assisted coiling	62 (40.8)

*SD, standard deviation; PcomA, posterior communicating artery; AcomA, anterior communicating artery; MCA, middle cerebral artery; PCA, posterior cerebral artery; PICA, posterior inferior cerebellar artery; AchA, anterior choroidal artery; BA, basilar artery; ICA, internal carotid artery*.

### Perioperative Complications

Among the patients, 18 (11.8%) experienced intra and/or postoperative complications. Intraprocedural re-rupture of the aneurysms occurred in two patients and was treated with rapid coiling. However, one of these patients suffered postoperative rebleeding and died, while the other showed no significant neurological deficit. Acute intraoperative thrombosis occurred in seven patients. All of them were treated with tirofiban thrombolysis and five of them were recanalized. In the other two patients, the thrombus did not dissolve and eventually led to postoperative cerebral infarction.

Intraprocedural coil migration occurred in two patients (1.3%) treated with CA and was managed conservatively without retrieval. The blood flow was not compromised at the end of the procedure. Neither of these patients showed neurological deficits at discharge. Four patients presented with postoperative rebleeding. In addition to the patient who suffered intraoperative and postoperative rebleeding, two patients were treated with extra-ventricular drainage and eventually discharged due to severe conditions. One patient was treated conservatively due to low bleeding and discharged with good recovery. Postoperative cerebral infarction occurred in six patients. One of them underwent bone flap decompressive craniectomy for a large cerebral infarction and was eventually lost to follow-up. The other five were treated with conservative medication and hyperbaric oxygen. Three of them recovered well, while the other two developed aphasia and hemiparesis in one limb.

A univariate analysis showed that there was no independent risk factor for the occurrence of perioperative complications. The incidence of perioperative complications in patients treated with SAC was higher than the incidence in those treated with CA, but the difference was not statistically significant (7.8 vs. 17.7%, *P* = 0.062; Table 2).

**Table 2:** Risk factors for procedure-related complications.

**Characteristics**	**Complications** **(*n* = 18)**	**No complications** **(*n* = 134)**	**Univariate *P*-value**
Age, mean ± SD, years	55.7 ± 12.7	56.2 ± 11.1	0.512
Female, *n* (%)	9 (50.0)	89 (66.4)	0.172
Hypertension, *n* (%)	9 (50.0)	64 (47.8)	0.858
Diabetes mellitus, *n* (%)	1 (5.6)	6 (4.5)	0.594
Smoking, *n* (%)	1 (5.6)	10 (7.5)	1.000
Hunt-Hess grade 1-2, *n* (%)	11 (61.1)	95 (70.9)	0.543
CT Fisher grade 1-2, *n* (%)	10 (55.6)	79 (59.0)	0.454
Aneurysm size, mean ± SD, mm	2.5 ± 0.42	2.5 ± 0.44	0.392
Neck size, mean ± SD, mm	2.1 ± 0.47	1.9 ± 0.54	0.344
Neck-dome ratio, mean ± SD	0.99 ± 0.27	0.89 ± 0.27	0.342
Anterior circulation, *n* (%)	16 (88.8)	129 (96.2)	0.852
Treatment modality			0.062
Coiling alone	7 (38.9)	83 (61.9)	
Stent-assisted coiling	11 (61.1)	51 (38.1)	

### Angiographic Results and Clinical Outcomes at Discharge and Follow-Up

**Figure 2:**
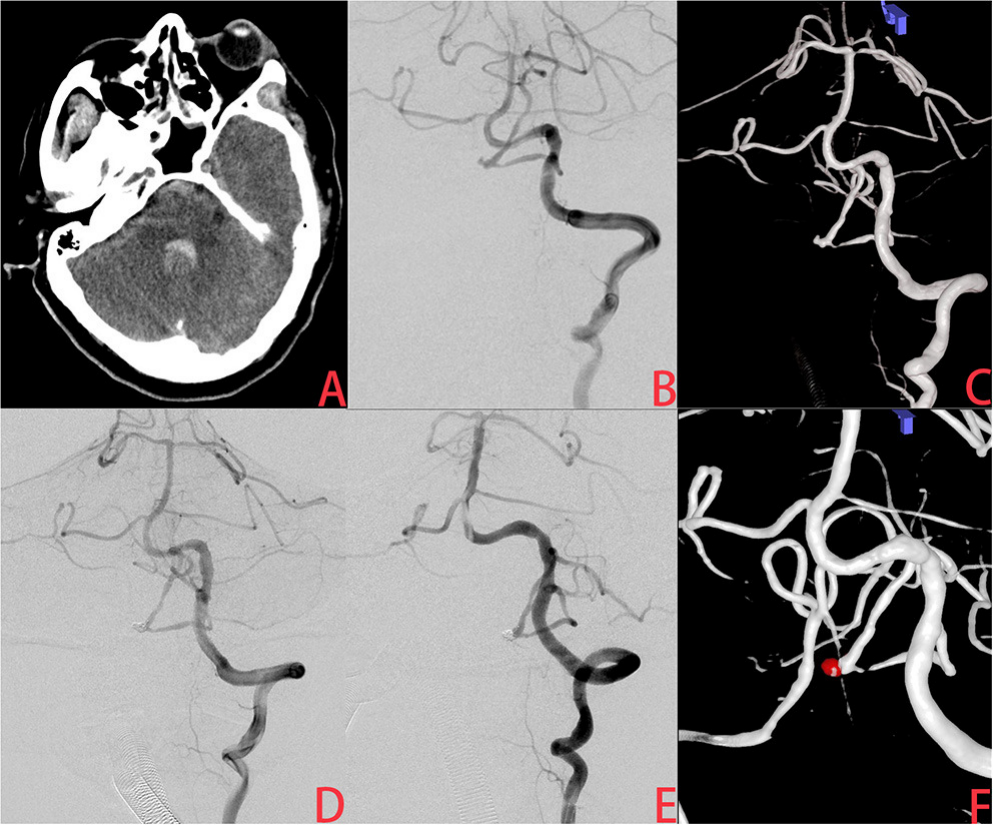
A ruptured very small posterior inferior cerebellar artery (PICA) intracranial aneurysm treated with coiling alone (CA). **(A)** The patient was admitted with spontaneous subarachnoid hemorrhage. **(B,C)** Cerebral angiography and 3D reconstruction revealed a tiny PICA aneurysm. **(D)** The aneurysm was treated with coiling embolization only. Immediate angiography showed that the aneurysm was completely occluded. **(E,F)** Six months later, angiographic images showed postoperative recurrence of the aneurysm.

The immediate angiographic results showed that complete occlusion (Raymond-Roy grade 1) was achieved in 116 (76.3%) patients, neck remnant (grade 2) was achieved in 27 (17.7%) patients, and partial occlusion (grade 3) was achieved in nine (6.0%) patients. A total of 105 patients were followed for 7.5 ± 6.8 months and evaluated with DSA. Among them, complete occlusion (grade 1) was achieved in 73, 23 had neck remnant (grade 2), and nine had partial occlusion (grade 3) (Table 3). Eighteen patients experienced postoperative aneurysm recurrence (Figure 2) and seven of them received retreatment with recurrence and retreatment rates of 17.1 and 6.7%, respectively. Of the 18 recurrent aneurysms, 17 were treated initially with CA and one was treated with SAC. The use of SAC embolization significantly reduced the recurrence rate of postoperative aneurysm. Univariate analysis showed that the use of stents was the only significant factor that affected the postoperative recurrence of aneurysms (18.8 vs. 1.6%, *P* < 0.001; Table 4).

**Table 3:** Aneurysm occlusion and clinical outcomes at discharge and follow-up.

**Outcomes**	**At discharge, *n* (%)**	**At follow-up, *n* (%)**
**Aneurysm occlusion (RR grade)**		
1	116 (76.3)	73 (69.5)
2	27 (17.7)	23 (21.9)
3	9 (6.0)	9 (6.0)
**Clinical outcomes (GOS score)**		
4-5	131 (86.2)	102 (97.1)
1-3	21 (13.8)	3 (2.9)

*RR, Raymond-Roy; GOS, Glasgow Outcome Scale*.

**Table 4:** Risk factors for aneurysm recurrence.

**Characteristics**	**Recurrent** **aneurysms** **(*n* = 18)**	**Stable** **aneurysms** **(*n* = 87)**	**Univariate** ***P*****-value**
Age, mean ± SD, years	53.6 ± 7.6	55.5 ± 10.5	0.076
Female, *n* (%)	13 (72.2)	59 (68.6)	0.762
Hypertension, *n* (%)	9 (50.0)	45 (52.3)	0.857
Diabetes mellitus, *n* (%)	0	4 (4.7)	1.000
Smoking, *n* (%)	1 (5.5)	7 (8.1)	0.602
Hunt-Hess grade1-2, *n* (%)	11 (61.1)	67 (77.9)	0.145
CT Fisher grade 1-2, *n* (%)	9 (50.0)	56 (65.1)	0.411
Aneurysm size, mean ± SD, mm	2.7 ± 0.36	2.6 ± 0.45	0.067
Neck size, mean ± SD, mm	1.8 ± 0.41	1.9 ± 0.58	0.075
Neck-dome ratio, mean ± SD	0.78 ± 0.18	0.93 ± 0.29	0.155
Anterior circulation, *n* (%)	16 (88.8)	83 (95.4)	0.477
Immediate aneurysm occlusion (mRS)			0.555
1	14 (77.8)	67 (77.9)	
2	3 (16.6)	13 (15.1)	
3	1 (5.6)	6 (7.0)	
Treatment modality			0.000
Coiling alone	17 (94.4)	42 (47.8)	
Stent-assisted coiling	1 (5.6)	45 (52.2)	

At discharge, 131 (86.2%) patients recovered well (GOS scores: 4–5) and 20 patients were left with various degrees of neurological deficits (morbidity rate = 13.1%). One patient died of intraoperative aneurysm rupture and postoperative rebleeding (mortality rate = 0.65%). A total of 105 patients were followed and 97.1% of them had good clinical outcomes (GOS scores ≥ 4) (Table 3). Univariate analysis showed that the preoperative Hunt-Hess grade, CT Fisher grade, and perioperative complications were independent risk factors for the clinical outcomes. Multivariate logistic regression analysis revealed that perioperative complication was the most significant influential factor on the clinical outcomes (*P* < 0.001; Table 5).

**Table 5:** Risk factors for clinical outcomes.

**Characteristics**	**GOS 4-5** **(*n* = 132)**	**GOS 1-3** **(*n* = 20)**	**Univariate** ***P*****-value**	**Multivariate** ***P*****-value**
Age, mean ± SD, years	55.6 ± 10.7	59.9 ± 14.1	0.051	-
Female, *n* (%)	86 (65.2)	12 (60.0)	0.654	-
Hypertension, *n* (%)	62 (47.0)	11 (55.0)	0.503	-
Diabetes mellitus, *n* (%)	7 (5.3)	0	0.595	-
Smoking, *n* (%)	10 (7.6)	1 (5.0)	1.000	-
Hunt-Hess grade 1-2, *n* (%)	97 (73.5)	9 (45.0)	0.019	0.169
CT Fisher grade 1-2, *n* (%)	83 (62.9)	6 (30.0)	0.038	0.353
Aneurysm size, mean ± SD, mm	2.58 ± 0.43	2.58 ± 0.46	0.751	-
Neck size, mean ± SD, mm	1.9 ± 0.54	2.0 ± 0.57	0.438	-
Neck-dome ratio, mean ± SD	0.89 ± 0.27	0.98 ± 0.25	0.563	-
Anterior circulation, *n* (%)	126 (95.4)	19 (95.0)	0.628	-
Treatment modality			0.165	-
Coiling alone	81 (61.4)	9 (45.0)		
Stent-assisted coiling	51 (38.6)	11 (55.0)		
Complications	9 (6.8)	9 (45.0)	0.000	0.000

## Discussion

In the present study, we retrospectively analyzed the clinical data and imaging results for 152 ruptured VSIAs treated with coil embolization and found that endovascular treatment was a safe and efficient approach for ruptured VSIAs. SAC embolization reduced the recurrence rate of postoperative aneurysm without increasing the incidence of perioperative complications. The preoperative Hunt-Hess grade, CT Fisher grade, and perioperative complications were significantly associated with the prognosis of patients with ruptured VSIAs. Perioperative complication was the most significant influential factor on the prognosis of these patients.

Treatments for VSIAs are often associated with increased preoperative complications, including IPR, due to the small cavity and frail wall of VSIAs (9). Rahmanian et al. (6) treated 32 VSIAs with the double-clip technique and the IPR rate was 30.8%. A meta-analysis of 1,295 aneurysms treated with coil embolization showed that the IPR rate in VSIAs was twice as high as that in larger aneurysms (7.7 vs. 3.6%; *P* = 0.018) (1). In a study by Nguyen et al. (10), the IPR rate in ruptured VSIAs during endovascular treatment was five times higher than that of other ruptured aneurysms. With the development of interventional materials and operation techniques, the IPR rate of endovascular treatment for VSIAs has decreased. A recent meta-analysis by Yamaki et al. (11) reported the IPR rate of endovascular treatment for VSIAs was 7%, which was lower than they had previously reported (8.3%) (7). In the study by Pop et al. (12), 97 patients with VSIAs were treated by coil embolization and only one patient experienced IPR (1%). In our cohort, perioperative aneurysm re-rupture occurred in only two patients (IPR rate = 1.3%), which was consistent with previous studies. For patients with IPR, protamine should be immediately administered to neutralize heparin, and the coils should be quickly filled to stop the bleeding. In addition, balloon placement has been shown to improve the prognosis of patients. Nguyen et al. found that when IPR of an aneurysm occurred, the clinical outcomes for patients who received balloon placement before embolization were significantly better than those for patients without balloon placement. When the aneurysm ruptures, filling the balloon can quickly stop the bleeding and prevent further increases in intracranial pressure. Surgeons may also have more time for coil packing or extra-ventricular drainage, which can improve the prognosis of the patient.

Since the majority of VSIAs are wide-necked aneurysms, SAC is often needed. However, in the acute stage of subarachnoid hemorrhage, the body is in a hypercoagulable state and, therefore, the use of stents may increase the risk of ischemic events. In addition, the use of dual-antiplatelet drugs after surgery may increase the risk of re-hemorrhage, especially for patients who need external ventricular drainage. Roh et al. (13) reported that the rate of thromboembolic complications in stent-assisted coil embolization of ruptured intracranial aneurysms was approximately twice as high as that in non-stent coil embolization, and external ventricular drainage-related re-hemorrhage was approximately five times higher than that of non-stent coil embolization. However, Xue et al. (14) compared the outcomes of acute ruptured intracranial aneurysms treated with low-profile visualized intraluminal support SAC or CA and found no significant difference in the overall incidence of complications between the two groups (7.2 vs. 4.3%, *P* = 0.207). In our study, univariate analysis showed that there was no significant risk factor for the overall perioperative procedure-related complications, including hemorrhagic complications and ischemic complications. Zuo et al. (15) also compared the incidence of procedure-related complications in patients treated with SAC or CA and found no significant difference between the two groups (8.3 vs. 4.5%, *P* = 0.120). Thus, they concluded that SAC was a safe, feasible, and promising treatment option for ruptured intracranial aneurysms. In the present study, the incidence of overall complications in the SAC group was higher than that in the CA group, but the difference was not significant (17.7 vs. 7.7%, *P* = 0.062). Univariate analysis showed that there was no significant risk factor for total perioperative procedure-related complications, which was consistent with previous studies.

Postoperative recurrence of aneurysms is a common complication that affects the long-term prognosis of patients. Zheng et al. (16) treated 501 wide-neck intracranial aneurysms with SAC embolization. The postoperative recurrence rate was 13.9% and the retreatment rate of aneurysms was 3.5%. Logistic analysis showed that larger aneurysm size and initial incomplete aneurysm occlusion were predictors of recanalization. Zhang et al. (17) treated 92 patients with ruptured VSIAs using CA or SAC, and the postoperative recurrence rate was 7.5%. Multivariate regression analysis showed that coiling without stents was the only factor significantly associated with aneurysm recurrence. Other studies also reported that SAC embolization significantly reduced the recurrence and retreatment rates of aneurysms (14, 18, 19). In this study, a total of 105 patients who received DSA were followed. Eighteen of them experienced postoperative recurrence (recurrence rate = 17.1%). Among them, seven received retreatment (retreatment rate = 6.7%). Univariate analysis showed that the use of stents was an independent factor that affected the postoperative recurrence of aneurysms, which was consistent with previous studies. Stents prevent the coil from protruding into the parent vessel. In addition, stent wires across the aneurysm neck reduce the impact of blood flow on the aneurysm and provide a structural basis for endothelialization, which may promote complete aneurysm occlusion and reduce aneurysm recurrence.

Safety evaluation is crucial for determining the therapeutic approach for ruptured intracranial aneurysms. Previous studies have identified the factors that affect the clinical prognosis of patients with ruptured intracranial aneurysms. Peng et al. (20) treated 272 ruptured small aneurysms (<5 mm in diameter) with coiling or SAC embolization, and found that preoperative Hunt-Hess grade, CT Fisher grade, and acute intraoperative thrombosis were independent factors affecting the clinical outcomes of patients. Li et al. (5) treated 162 ruptured VSIAs with coil embolization or microsurgical clipping. Multiple regression analysis showed that age, Hunt-Hess grade, cerebral vasospasm, and perioperative complications were significantly associated with the clinical outcomes of patients no matter the treatment method adopted. In the present study, univariate analysis revealed that the preoperative Hunt-Hess grade, CT Fisher grade, and perioperative complications were independent risk factors for the clinical prognosis of patients. Further multiple logistic regression analysis showed that perioperative complication was the most significant factor, which was in line with previous studies.

There were several limitations to the present study. Firstly, there may be some selection bias due to the retrospective nature of the study. Secondly, the sample size was small, and the duration of follow-up was short. A prospective study with a larger sample size and longer follow-up is needed to further explore the safety and efficiency of CA and stent-assisted coil embolization for the treatment of ruptured VSIAs.

Endovascular treatment is a safe and efficient approach for ruptured VSIAs. SAC reduces the recurrence rate of aneurysms without increasing the incidence of perioperative complications. The Hunt-Hess grade, CT Fisher grade, and perioperative complications are independent factors that affect the clinical outcomes of patients with ruptured VSIAs, and perioperative complication is the most significant influential factor.

## Data Availability Statement

The original contributions presented in the study are included in the article/supplementary material, further inquiries can be directed to the corresponding author/s.

## Ethics Statement

The study protocol was approved by the Ethics Committee of the Yijishan Hospital Affiliated to Wannan Medical College. Written informed consent for participation was not required for this study in accordance with the national legislation and the institutional requirements.

## Author Contributions

FQ performed the studies and wrote the manuscript. JL and XZ performed the data analysis. DW and NL participated in drawing the images. ZZ and ZL contributed to the design and analysis of the study. All authors contributed to the article and approved the submitted version.

## Funding

This work was supported by Key project of Anhui Provincial Education Department (No. KJ2019A0423), the Science Research Project of Professional of the First Affiliated Hospital of Wannan Medical College (No. YR202004), and Anhui Province's Natural Science Foundation (No. 2108085QH328).

## Conflict of Interest

The authors declare that the research was conducted in the absence of any commercial or financial relationships that could be construed as a potential conflict of interest.

## Publisher's Note

All claims expressed in this article are solely those of the authors and do not necessarily represent those of their affiliated organizations, or those of the publisher, the editors and the reviewers. Any product that may be evaluated in this article, or claim that may be made by its manufacturer, is not guaranteed or endorsed by the publisher.

## References

[1] van Rooij WJ Keeren GJ Peluso JP Sluzewski M. Clinical and angiographic results of coiling of 196 very small (< or = 3 mm) intracranial aneurysms. AJNR Am J Neuroradiol. (2009) 30:835–9. 10.3174/ajnr.A1429 19131407 PMC7051764

[2] Weir B Disney L Karrison T. Sizes of ruptured and unruptured aneurysms in relation to their sites and the ages of patients. J Neurosurg. (2002) 96:64–70. 10.3171/jns.2002.96.1.0064 11794606

[3] Gupta V Chugh M Jha AN Walia BS Vaishya S. Coil embolization of very small (2 mm or smaller) berry aneurysms: feasibility and technical issues. AJNR Am J Neuroradiol. (2009) 30:308–14. 10.3174/ajnr.A1374 19001535 PMC7051409

[4] Malhotra A Wu X Forman HP Matouk CC Gandhi D Sanelli P. Management of tiny unruptured intracranial aneurysms: a comparative effectiveness analysis. JAMA Neurol. (2018) 75:27–34. 10.1001/jamaneurol.2017.3232 29159405 PMC5833486

[5] Li J Su L Ma J Kang P Ma L Ma L. Endovascular coiling versus microsurgical clipping for patients with ruptured very small intracranial aneurysms: management strategies and clinical outcomes of 162 cases. World Neurosurg. (2017) 99:763–9. 10.1016/j.wneu.2015.11.079 26732968

[6] Rahmanian A Ghaffarpasand F Alibai E Choque-Velasquez J Jahromi BR Hernesniemi J. Surgical outcome of very small intracranial aneurysms utilizing the double clip technique. World Neurosurg. (2018) 110:e605–11. 10.1016/j.wneu.2017.11.060 29162525

[7] Brinjikji W Lanzino G Cloft HJ Rabinstein A Kallmes DF. Endovascular treatment of very small (3 mm or smaller) intracranial aneurysms: report of a consecutive series and a meta-analysis. Stroke. (2010) 41:116–21. 10.1161/STROKEAHA.109.566356 19926837

[8] Raymond J Guilbert F Weill A Georganos SA Juravsky L Lambert A. Long-term angiographic recurrences after selective endovascular treatment of aneurysms with detachable coils. Stroke. (2003) 34:1398–403. 10.1161/01.STR.0000073841.88563.E9 12775880

[9] Signorelli F Scholtes F Bojanowski MW. [Very small intracranial aneurysms: clip or coil]. Neurochirurgie. (2012) 58:156–9. 10.1016/j.neuchi.2012.03.004 22481028

[10] Nguyen TN Raymond J Guilbert F Roy D Bérubé MD Mahmoud M. Association of endovascular therapy of very small ruptured aneurysms with higher rates of procedure-related rupture. J Neurosurg. (2008) 108:1088–92. 10.3171/JNS/2008/108/6/1088 18518708

[11] Yamaki VN Brinjikji W Murad MH Lanzino G. Endovascular treatment of very small intracranial aneurysms: meta-analysis. AJNR Am J Neuroradiol. (2016) 37:862–7. 10.3174/ajnr.A4651 26721770 PMC7960320

[12] Pop R Aloraini Z Mihoc D Burta H Manisor M Richter JS. Embolization of very small (≤3 mm) unruptured intracranial aneurysms: a large single-center experience on treatment of unruptured versus ruptured cases. World Neurosurg. (2019) 128:e1087–95. 10.1016/j.wneu.2019.05.070 31103760

[13] Roh H Kim J Bae H Chong K Kim JH Suh SI. Comparison of stent-assisted and no-stent coil embolization for safety and effectiveness in the treatment of ruptured intracranial aneurysms. J Neurosurg. (2019) 30:1–7. 10.3171/2019.5.JNS19988 31470411

[14] Xue G Zuo Q Tang H Zhang X Duan G Feng Z. Comparison of low-profiled visualized intraluminal support stent-assisted coiling and coiling only for acutely ruptured intracranial aneurysms: safety and efficacy based on a propensity score-matched cohort study. Neurosurgery. (2020) 87:584–91. 10.1093/neuros/nyaa110 32415845

[15] Zuo Q Yang P Lv N Huang Q Zhou Y Zhang X. Safety of coiling with stent placement for the treatment of ruptured wide-necked intracranial aneurysms: a contemporary cohort study in a high-volume center after improvement of skills and strategy. J Neurosurg. (2018) 131:435–41. 10.3171/2018.3.JNS172199 30117764

[16] Zheng Y Song Y Liu Y Xu Q Tian Y Leng B. Stent-assisted coiling of 501 wide-necked intracranial aneurysms: a single-center 8-year experience. World Neurosurg. (2016) 94:285–95. 10.1016/j.wneu.2016.07.017 27424472

[17] Zhang Y Yang M Zhang H Zhang X Li Y Jiang C. Stent-assisted coiling may prevent the recurrence of very small ruptured intracranial aneurysms: a multicenter study. World Neurosurg. (2017) 100:22–9. 10.1016/j.wneu.2016.12.107 28062369

[18] Zhang X Zuo Q Tang H Xue G Yang P Zhao R. Stent assisted coiling versus non-stent assisted coiling for the management of ruptured intracranial aneurysms: a meta-analysis and systematic review. J Neurointerv Surg. (2019) 11:489–96. 10.1136/neurintsurg-2018-014388 30842307

[19] Froelich JJ Cheung N de Lange JA Monkhorst J Carr MW DeLeacy R. Residuals, recurrences and re-treatment after endovascular repair of intracranial aneurysms: a retrospective methodological comparison. Interv Neuroradiol. (2020) 26:45–54. 10.1177/1591019919867841 31403834 PMC6997995

[20] Peng F Feng X Tong X Zhang B Wang L Guo E. Endovascular treatment of small ruptured intracranial aneurysms (<5 mm) : long-term clinical and angiographic outcomes and related predictors. Clin Neuroradiol. (2020) 30:817–26. 10.1007/s00062-019-00835-8 31696281 PMC7728636

